# Frontal Headache and Iodide of Potash

**Published:** 1893-09

**Authors:** 


### Frontal Headache and Iodide of Potash.

A heavy, dull headache, situated over the brow, and accompanied by languor, chilliness and a feeling of general discomfort, with distaste for food, which sometimes approaches to nausea, can generally be completely removed by a two-grain dose of the potassic salt dissolved in half a wine glass of water, and this quietly sipped, the whole quantity being taken in about ten minutes. In many cases the effect of these small doses has been simply wonderful. A person who, a quarter of an hour before, was feeling most miserable and refused all food, wishing only for quietness, would now take a good meal and resume his wonted cheerfulness. The rapidity with which the iodide acts in these cases constitutes its great advantage.—*Alienist and Neurologist.*

